# Migraine treatment: a chain of adverse effects

**DOI:** 10.1186/s40064-015-1206-1

**Published:** 2015-08-11

**Authors:** Tiago Sousa Veloso, Mariana Seixas Cambão

**Affiliations:** Family Medicine Resident, Garcia de Orta Family Health Unit, West Porto Group of Health Centres, Porto, Portugal; Neurosciences and Mental Health Department, Medical Psychology Unit, Faculty of Medicine of the University of Porto, Porto, Portugal; Family Medicine Resident, Ramalde Family Health Unit, West Porto Group of Health Centres, Porto, Portugal

**Keywords:** Migraine disorders (MeSH term), Inappropriate prescribing (MeSH term), Drug-related side effects and adverse reactions (MeSH term), Diskinesia, drug-induced (MeSH term), Quaternary prevention, Prescribing cascade

## Abstract

This clinical vignette presents a 14 years old female, with a past medical history relevant only for migraine with typical aura of less than monthly frequency, complaining of a severe unilateral headache with rising intensity for the previous 4 h, associated with nausea, vomiting, photophobia and phonophobia. This episode of migraine with aura in a patient with recurrent migraine was complicated by side effects of medical diagnostic and therapeutic procedures (extrapyramidal symptoms, delirium, post-lumbar puncture headache, hospital admission) all of which could have been prevented—quaternary prevention. This case illustrates several important messages in migraine management: (1) use of acetaminophen is not based in high-quality evidence and better options exist; (2) among youngsters, domperidone should be preferred over metoclopramide because it does not cross the blood–brain barrier; (3) moderate to severe migraine crisis can be managed with triptans in teenagers over 12 years old; (4) it is important to recognize adverse drug effects; (5) harmful consequences of medical interventions do occur; (6) the school community must be informed about chronic diseases of the young.

## Background

*Primum non nocere* is a cornerstone principle of *ars medica* (medical practice). Harmful consequences can derive from everyday drug treatments and it is only through a high-degree of suspicion that certain etiological diagnosis can be made. Prescribing cascades and careless use of ancillary studies can only be avoided if adverse drug effects are foreseen and identified—quaternary prevention. This case illustrates several important messages in quaternary prevention during migraine management.

Headaches are a common complaint among adolescents (from 8 to 23 %) (Lewis et al. 2004) and it is estimated that two-thirds of sufferers are managed in primary care (American Headache Society and Primary Care Migraine Partnership 2004). Among the main types of primary headache, migraine is the leading cause of disability, and alone is itself the eighth major cause of disability among both men and women worldwide (Vos et al. 2010).

The clinical vignette here presented pictures the harmful effects that may derive from medical interventions and how they may change the natural history of migraine. It is important not only to recognize disease but also to anticipate and consider potential adverse effects of medical practice in order to avoid harming patients (*primum non nocere*).

## Case presentation

A 14-year-old girl presented to her primary care center with a severe unilateral headache that had progressed over a 4 h period from typical aura to moderate pain with photophobia and phonophobia to vomiting and to high-intensity headache. Her relevant past medical history included Migraine with Typical Aura (<1 per month) diagnosed at 12 years old after excluding major organic disease. The physical examination was notable for the normal blood pressure and temperature, absence of neurologic deficits and meningeal signs, as well as the absence of any other sign or symptom suggestive of a secondary headache. At this time, the whole clinical picture was compatible with an increased intensity flare of the previously diagnosed migraine with typical aura due to her lack of opportunity to administer herself the abortive therapy at the appropriate moment in time (earlier that morning, during a school class, she had felt the beginning of the aura; unfortunately, her teacher did not allow her to leave the room in order to have access to the abortive treatment).

At the primary care center, she received intravenous acetaminophen (paracetamol) and metoclopramide. No more than half an hour had passed when she started developing dystonia of the upper limbs. No improvement of her presenting headache was noted. Decision was made of transferring her to the Pediatrics emergency unit for a diagnostic and therapeutic procedure. After admission to the hospital, these extrapyramidal symptoms were managed with biperiden (a centrally acting antiparkinsonian anticholinergic drug). A few minutes later the teenager became confused, spatially disoriented and with short-term memory disturbance. This acute confusional state lead to a diagnostic procedure which included a lumbar puncture and the consideration of an electroencephalogram, although not immediately available. After disclosure of the laboratory results, which were entirely unremarkable, the patient remained under medical supervision for several hours and the delirium gradually faded away. This evolution helped reinforcing the etiological role of biperiden. During this time, a spontaneous improvement from the migraine was noted as well as total recovery of orientation and confusion. Later on, after admission to the Pediatrics ward, the patient complained of another headache, but this time with a different presentation (frontal pain, associated with the upright position, relieved by lying down). A post-lumbar puncture headache was diagnosed, which prolonged her hospital stay for over 48 h and her home rest and school absence for the following 5 days. At the time of hospital discharge a neurology consultation was scheduled in order to decide for the need of further diagnostic study for the migraine. At that neurology appointment, a brain magnetic resonance imaging (MRI) scan was requested (recall that 2 years before, at her 12-years-old, a diagnostic procedure for a recurrent headache, including a brain computerized tomography (CT) scan, had been done and supported the migraine diagnosis). Indeed, the brain MRI turned out to be, not surprisingly, unremarkable.

## Discussion

This case illustrates several important messages in migraine management.

1. The use of acetaminophen as migraine abortive (symptomatic) therapy has limited evidence of efficacy and is not a first-line treatment in major national (Pereira-Monteiro 2009; Portuguese Headache Society 2010) nor international (Steiner et al. 2007, 2011) guidelines. Acetaminophen use did not even reduce the need for rescue medication when compared to placebo (Damen et al. 2005). Alternative drugs exist supported by high-quality evidence (at least one randomized-controlled trial and low alpha and beta types of error)—for instance: ibuprofen or acetylsalicylic acid (the latter is not intended for use in people under 12 or under 16 if feverish, although the relationship between aspirin intake and Reye syndrome is not supported by sufficient facts) (Schor 2007). Moreover, intravenous acetaminophen has not been evaluated in randomized controlled trials in the pediatric population, and has shown not to be superior to placebo in treating severe acute migraine attacks in adults (Leinisch et al. 2005).
2. Among youngsters, domperidone should be preferred over metoclopramide (Pereira-Monteiro 2009) because it does not cross the blood–brain barrier (Teixeira 2001). Metoclopramide was even associated with a 31% increased odds of emergency department revisit than other antiemetic drugs (Bachur et al. 2015). Domperidone is the only antiemetic drug licensed for use in children up to 12 years of age (Evers et al. 2009).
3. Moderate to severe migraine crisis in teenagers over 12 years old can be managed with sumatriptan (Winner et al. 2000; Curran et al. 2005; Cady and Schreiber 2006; Evers et al. 2009, Pereira-Monteiro 2009; Goldman and Meckler 2015), rizatriptan (Ahonen et al. 2006) or zolmitriptan (Evers et al. 2006; Lewis et al. 2007) and the efficacy of these drugs has been supported by moderate to high quality evidence. The off-label use of triptans is still a matter of debate, however more recently sumatriptan nasal spray and zolmitriptan nasal spray have been approved for adolescents in Europe; almotriptan has been approved for adolescents in the USA, as has rizatriptan for patients aged 6–17 years (Wöber-Bingöl 2013).
4. Not only is it important to be familiar with the most common adverse effects of drugs prescribed (Garjón 2011), but it is a matter of utmost importance being able to recognize them. Mastering dosing, adverse effects and interactions will place physicians in a better position to prevent errors and anticipate problems (Schiff et al. 2011). Whenever possible, educate patients about possible adverse effects to ensure that they are recognized as early as possible (Schiff et al. 2011).
5. Harmful consequences of medical interventions do occur, deteriorate patients’ quality of life, make diagnosis and management of patients’ disease more difficult, and can lead to what is known as “prescription cascade”, that is, treatment of the undesirable effect of a drug by another drug (Garjón 2011). A major prescribing cascade scenario has been illustrated in this clinical report: biperiden, as a muscarinic receptor antagonist, was used to control the extrapyramidal motor symptoms (Brocks 1999) induced by metoclopramide. Biperiden can be administered through oral, intravenous and intramuscular routes in dosages starting from 1 to 2 mg per day up to 12 mg/day (in 2 or 3 separate doses) or even higher (doses of 40 mg/day have been reported in severe dystonia patients) (Oztekin et al. 1991). Toxicity is mainly dependent on its anticholinergic properties, and instead of others of its class, no reports of fatalities due to biperiden seem to be available (Gjerden and Slørdal 1998). Reviewing current drug therapy is an essential component of medical care. Therefore, careful consideration should be given to any new symptom in order to decide whether the development of a new medical condition could be the presentation of an atypical adverse drug effect to an existing drug therapy (Rochon 2014). Moreover, if we enlarge the concept of prescribing cascade and allow it to include a diagnostic domain, then one *diagnostic* prescribing cascade is also illustrated by this clinical vignette: the acute confusional state induced by biperiden, resulting from its anticholinergic properties (Standaert and Young 2001), was conducive to a broader ancillary work-up, including a lumbar puncture (which in this case became complicated by a post-lumbar puncture headache) and a brain MRI.
6. Last but not least, it is necessary to inform and involve the school community in the care of children and teenagers with chronic diseases as this may positively contribute to better health outcomes (Yonker 2009). This case report illustrates how a seemingly unimportant authorization for leaving the class (in order to have access to a glass of water and some drug treatment) could have avoided the escalating severity of headache and the undesirable related morbidity.

The optimal treatment of migraine must be customized to the patient and to the attack. Patient empowerment must be promoted to enhance personal control of migraine, to reduce use of resources, to rapidly restore ability to function, to improve patient satisfaction and to minimize use of back-up and rescue medications (Lewis et al. 2004). Should a migraine episode develop to a full-blown crisis, a stratified care approach is advisable (as opposed to a stepped care approach) (Lipton 1998; Rothrock 2012) which will guide management according to peak intensity, time to peak intensity, any associated symptoms and disability. The pharmacological treatment of the status migrainosus may include acetaminophen, a non-steroidal anti-inflammatory drug (e.g. ibuprofen or naproxen), a triptan (consider the nasal route when emetic symptoms are present), dihydroergotamine, selected opioids and selected corticosteroids. For many children, merely inducing sleep is effective. Anti-emetics should also be considered as necessary.

## Conclusions

This case illustrates several important messages in quaternary prevention during migraine management: (1) use of acetaminophen is not based in high-quality evidence and better options exist; (2) among youngsters, domperidone should be preferred over metoclopramide because it does not cross the blood–brain barrier; (3) moderate to severe migraine crisis can be managed with triptans in teenagers over 12 years old; (4) it is important to recognize adverse drug effects; (5) harmful consequences of medical interventions do occur; (6) the school community must be informed about chronic diseases of the young.

## Consent

Written informed consent was obtained from the parents of the patient for publication of this Case report. A copy of the written consent is available for review by the Editor-in-Chief of this journal.

## Abbreviations

CT: computerized tomography
MRI: magnetic resonance imaging
